# Association of lung function with overall mortality is independent of inflammatory, cardiac, and functional biomarkers in older adults: the ActiFE-study

**DOI:** 10.1038/s41598-020-68372-w

**Published:** 2020-07-17

**Authors:** Gudrun Weinmayr, Holger Schulz, Jochen Klenk, Michael Denkinger, Enric Duran-Tauleria, Wolfgang Koenig, Dhayana Dallmeier, Dietrich Rothenbacher, The ActiFE Study Group, B. Böhm, H. Geiger, R. Laszlo, J. M. Steinacker, A. Ludolph, C. von Arnim, A. Lukas, R. Peter, K. Rapp, M. Riepe, K. Scharffetter-Kochanek, J. Stingl

**Affiliations:** 1 Institute of Epidemiology and Medical Biometry, Ulm University, Helmholtzstraße 22, 89081 Ulm, Germany; 2 Institute of Epidemiology, Helmholtz Zentrum München – German Research Center for Environmental Health, Neuherberg, Germany; 3 Comprehensive Pneumology Center Munich (CPC-M), Member of the German Center for Lung Research (DZL), Munich, Germany; 4 Department of Geriatrics and Geriatric Rehabilitation, Robert-Bosch-Hospital, Stuttgart, Germany; 5 IB Hochschule Berlin, Studienzentrum Stuttgart, Stuttgart, Germany; 6 Agaplesion Bethesda Clinic, Geriatric Research Unit, Ulm University, Ulm, Germany; 7 Geriatric Center Ulm/Alb-Donau, Ulm, Germany; 8 Global Institute of Neurodevelopment Integrated Care (IGAIN), Barcelona, Spain; 9 Deutsches Herzzentrum München, Technische Universität München, Munich, Germany; 10 DZHK (German Centre for Cardiovascular Research), Partner Site Munich Heart Alliance, Technische Universität München, Munich, Germany; 11 Department of Epidemiology, Boston University School of Public Health, Boston, USA; 12 Lee Kong Chian School of Medicine, Nanyang Technological University, Singapore, Singapore; 13 Imperial College London, London, UK; 14 Institute for Molecular Medicine, Ulm University, Ulm, Germany; 15 Department of Internal Medicine II - Sports- and Rehabilitation Medicine, Ulm University Medical Center, Ulm, Germany; 16 Department of Neurology, Ulm University Medical Center, Ulm, Germany; 17 Malteser Krankenhaus Seliger Gerhard Bonn/Rhein-Sieg, Bonn, Germany; 18 Institute of the History, Philosophy and Ethics of Medicine, Ulm University, Ulm, Germany; 19 Division of Gerontopsychiatry, Department of Psychiatry and Psychotherapy II, Ulm University Medical Center, Ulm, Germany; 20 Department of Dermatology and Allergology, Ulm University Medical Center, Ulm, Germany; 21 Department of Pharmacology, University of Aachen, Aachen, Germany

**Keywords:** Predictive markers, Cardiology, Respiratory tract diseases, Epidemiology, Risk factors

## Abstract

Reduced lung function is associated with overall and cardiovascular mortality. Chronic low grade systemic inflammation is linked to impaired lung function and cardiovascular outcomes. We assessed the association of lung function with overall 8-year mortality in 867 individuals of the Activity and Function in the Elderly study using confounder-adjusted Cox proportional hazards models (including gait speed and daily walking time as measures of physical function) without and with adjustment for inflammatory and cardiac markers. Forced expiratory volume in 1 s/forced vital capacity (FEV_1_/FVC) but not FVC was related to mortality after adjustment for physical function and other confounders. Additional adjustment for inflammatory and cardiac markers did not change the hazard ratios (HR) markedly, e.g. for a FEV_1_/FVC below 0.7 from 1.55 [95% confidence-interval (CI) 1.14–2.11] to 1.49 (95% CI 1.09–2.03). These independent associations were also observed in the apparently lung healthy subpopulation with even higher HRs up to 2.76 (95% CI 1.52–4.93). A measure of airflow limitation but not vital capacity was associated with overall mortality in this community-dwelling older population and in the subgroup classified as lung healthy. These associations were independent of adjustment for inflammatory and cardiac markers and support the role of airflow limitation as independent predictor of mortality in older adults.

## Introduction

Pulmonary and cardiovascular diseases are important determinants of morbidity, disability and mortality in older adults. Therefore, surrogate markers of high risk for adverse health outcomes for these disease entities may be useful components of risk assessment and risk stratification to target preventive measures. A link between poor lung function and all-cause mortality has also been observed in individuals without any manifest lung disease. However, it is not entirely clear, which lung function parameters are most related to mortality^1–3^, especially in older populations.

Reduced lung function and especially chronic obstructive pulmonary disease (COPD) have been linked to chronic low grade systemic inflammation, which at the same time is a key driver of atherosclerotic cardiovascular disease (CVD) and cardiovascular ageing^4,5^. Inflammatory markers such as Interleukin 6 (IL-6) and C-reactive protein (CRP) are associated with lung function^6^, endothelial dysfunction and atherosclerosis^7^. In addition, mortality associated with reduced lung function may be partly linked to a concomitantly impaired cardiac function, especially in older adults. In patients with COPD and chronic respiratory failure, 13% were reported to die of heart failure (HF) in a large European study^8^. Furthermore, people with mild to moderate COPD die more often of cardiovascular causes than of respiratory failure^9^. The significance of cardiovascular limitations is supported by the observation that high-sensitive cardiac troponin T (hscTnT), a marker of myocardial injury, has been associated with impaired lung function in a population of older men^10^ as has been N-terminal pro-brain natriuretic peptide (NT-proBNP), a marker of hemodynamic stress characterizing myocardial function.

To date, comprehensive analyses of the relation between lung function and mortality and the role of systemic inflammation and cardiac impairment therein are still rare for the general older population. Therefore, we investigated the relation of various lung function parameters with all-cause mortality in a large cohort study of community dwelling older adults and analysed whether this relation is independent of biomarkers of inflammation, cardiac function and myocardial injury.

## Methods

A further detailed description is given in the Supplementary Information online.

### Study population

The study protocol of the Activity and Function in the Elderly in Ulm (ActiFE-Ulm) study^11^, including details of recruitment and the study population, has been reported elsewhere^12,13^. In brief, a population-based random sample of community-dwelling older persons (≥ 65 years) in Ulm, Southern Germany, and adjacent areas was contacted and 1506 individuals were recruited at baseline.

The aim of the ActiFE-Ulm study was “to identify (modifiable) risk factors for the onset of functional decline or disabilities, to provide new answers to overcome these causes of incident disabilities but also to identify resources of healthy ageing”^11^. One main focus was the influence of physical activity (PA) on these parameters. The Indicators for Monitoring COPD and Asthma (IMCA) II project funded by the EU, was conducted to get information on the importance of respiratory indicators using questionnaire items and lung function testing in the elderly. Because of resulting synergies allowing a careful and extensive characterization of the study population, these two initiatives were combined in Ulm and carried out in the same study population resulting in the ActiFE-Ulm study.

Participants underwent an extensive, standardized baseline interview and examination between March 2009 and April 2010. Blood samples were taken and subjects wore an uni-axial accelerometer (activPAL, PAL Technologies, Glasgow, Scotland) for 7 consecutive days (24 h per day) to measure PA^12^. All participants provided written informed consent and the research was carried out in accordance with relevant ethical guidelines and regulations. The Ethics Committee of Ulm University had approved the study (application no. 318/08 and 50/12).

### Spirometry

For 1,292 participants spirometry data were available as part of the IMCA II study^11^. Lung function measurements based on a forced expiratory flow manoeuvre were performed with EasyOne Spirometers (NDD, Switzerland) according to American Thoracic Society (ATS)/European Respiratory Society (ERS) criteria. The best three spirometry manoeuvres were retained for choosing the best FEV_1_ and FVC after a minimum of three manoeuvres (to a maximum of eight manoeuvres if necessary and feasible). Recordings were visually inspected for quality assessment of the spirometry. Of the reproducible manoeuvres (< 0.2L difference between two acceptable manoeuvres), the largest forced expiratory volume in 1 s (FEV_1_) and forced vital capacity (FVC) were chosen, respectively. Only pre-bronchodilator values were used in the current analysis.

### Laboratory measurements

In brief, high-sensitivity CRP (hsCRP) was measured with a high-sensitivity assay on a Behring Nephelometer II and IL-6 by ELISA with ELISA Quantikine HS, R&D. HscTnT and NT-proBNP were measured by electrochemoluminescence on an Elecsys 411 (Roche Diagnotics). High-sensitive troponin I (hscTnI) was measured on an ARCHITECT STAT (Abbott Diagnostics). Total cholesterol and high-density and low-density lipoprotein (HDL, LDL) cholesterol (homogeneous method) were measured spectrophotometrically on a Dimension RxL Max Integrated Chemistry System (Siemens) on the basis of a reaction catalyzed by cholesterol oxidase.

### Covariates

Age, sex, school education, smoking, environmental tobacco smoke (ETS), co-morbidity, intake of respiratory medication, occupational exposures (dust, chemicals, vapours) and exposure to traffic at home were ascertained by interview-based self-report. Height, weight and gait speed were measured. Average daily walking duration was calculated from accelerometer data for each individual^12^.

### Ascertainment of deaths

Vital status and death date were obtained from local registration offices for all participants. Censoring date was 18th January 2018. Time to death or censoring was calculated for each participant.

### Statistical analysis

Correlations between lung function parameters and clinical markers were assessed with Spearman rank correlation coefficients adjusted for age and sex.

Cox proportional hazards regression models with age as the underlying time variable^14^ were used to estimate the association between lung function (FEV_1_, FVC and FEV_1_/FVC as continuous variables and a dichotomous variable for FEV_1_/FVC with cut-point 0.7) and eight-year mortality. In a sensitivity analysis, we used percent predicted values based on equations of García-Río et al.^15^ (population in same age range) and Karrasch et al.^16^ (geographically close population). The base model was adjusted for sex and height in addition to age.

The following covariates were investigated as potential confounders: duration of school education (as proxy for socioeconomic status), body mass index (BMI), weight, sensor-based PA measured as daily walking time, smoking, ETS, occupational exposure to dust and chemicals, traffic exposure at home, serum-cholesterol (including HDL and LDL cholesterol), self-reported current intake of respiratory medication, and gait speed. Only covariates associated with mortality were retained for handwise forward selection (details for selection see Supplementary Information online). In this procedure only gait speed, daily walking time, intake of respiratory medication and HDL-cholesterol changed the log hazard ratio by 5% or more for any of the continuous lung function measures and were adjusted for in the main model.

To this main model, clinical biomarkers (hsCRP and IL-6, NT-proBNP, troponins) were introduced after natural-log-transformation. We tested for interaction of single biomarkers with each of the lung function parameters by introducing an interaction term into the model. All analyses were conducted using Stata software (version 14; StataCorp, College Station, TX, USA) except for adjusted spearman correlations, which were calculated with SAS 9.4 (SAS Institute Inc., Cary, NC, USA).

Regression models were performed for the total study population and in the apparently lung healthy part of the study population defined as follows: FEV_1_/FVC ≥ lower limit of normal (LLN) of predicted value according to García-Río et al.^15^, no self-reported diagnoses of COPD, asthma, chronic bronchitis or emphysema, no self-reported respiratory symptoms (wheeze, cough and phlegm over at least 3 months per year), no self-reported current intake of respiratory medication, no current smoker. The equations from García-Río et al*.*^15^ were chosen because of the comparable age range and FEV_1_/FVC ≥ LLN rather than the fixed ratio due to its higher specificity in an older population^17^ to retain most of the healthy individuals for this analysis.

## Results

### Subjects

Of the 1,064 individuals with reproducible spirometry, 867 (81%) had data available on all covariates and biomarkers and form the study population for the present analysis. Highest loss of participants was due to invalid accelerometer measured walking time (loss of 146 subjects (14%)). The final study population of 867 individuals had a lower eight-year mortality rate [28.4 per 1,000 person years (95% confidence interval (CI): 24.6–32.8)] than the total study population [N = 1506, mortality rate: 33.0 (95% CI 29.8–36.6) but was well comparable to the 1,064 individuals with reproducible spirometry [mortality rate of 29.8 (95% CI 26.3–33.9)].

Mean age of the final study population was 75.2 years, 42.7% were women, 44.9% ex-smokers and 5.9% current smokers (Table 1). 338 (39%) of those 867 individuals had a FEV_1_/FVC < 0.7, the cut-point introduced by the Global Strategy for the Diagnosis, Management, and Prevention of Chronic Obstructive Pulmonary Disease^18^. In comparison to the whole final study population and the apparently respiratory healthy group, the group with a FEV_1_/FVC < 0.7 was slightly older and had a higher percentage of men, ex-smokers, current smokers, self-reported intake of respiratory medication, COPD, asthma, myocardial infarction and, to a lesser extent, HF. Cardiac markers were higher (especially NT-proBNP and hscTnT), whereas inflammatory markers were not. Mortality rate per 1,000 person years was also highest with 45.3 (95% CI 37.6–54.7).

**Table 1:** Participant characteristics.

	Whole study population	Apparently lung healthy individuals^a^	Individuals with FEV_1_/FVC < 0.7
Number	867	428	338
Age years	75.2 (6.5)	74.4 (6.3)	76.9 (6.7)
Women	370 (42.7%)	186 (43.5%)	117 (34.6%)
School education ≤ 9 years	359 (41.8%)	172 (40.2%)	140 (41.4%)
Height m	1.67 (0.09)	1.67 (0.09)	1.68 (0.08)
BMI kg m^−2^	27.7 (4.1)	27.6 (4.1)	27.2 (3.9)
Non-smoker	426 (49.2%)	238 (55.6%)	132 (39.1%)
Ex-smoker	389 (44.9%)	190 (44.4%)	178 (52.7%)
Smoker	51 (5.9%)	–	28 (8.3%)
Exposure to ETS	49 (5.7%)	20 (4.7%)	15 (4.5%)
Respiratory symptoms in past year	140 (16.4%)	–	62 (18.6%)
Intake of respiratory medication	63 (7.3%)	–	44 (13.0%)
History of asthma	48 (5.5%)	–	33 (9.8%)
History of COPD	22 (2.6%)	–	19 (5.7%)
History of myocardial infarction	75 (8.7%)	32 (7.5%)	40 (11.8%)
History of heart failure	1,265 (14.5%)	48 (11.2%)	54 (16.0%)
History of hypertension	454 (52.4%)	220 (51.4%)	180 (53.3%)
History of diabetes	120 (13.8%)	56 (13.1%)	42 (12.4%)
Diabetes medication	93 (10.7%)	46 (10.8%)	33 (9.8%)
Statin use	217 (25.0%)	99 (23.1%)	93 (27.5%)
Habitual walking speed m s^−1^ (3 m)^b^	0.89 (0.32)	0.89 (0.31)	0.87 (0.35)
Habitual walking speed m s^−1^ (4 m)^b^	1.01 (0.28)	1.04 (0.27)	0.97 (0.28)
Walking duration min day^−1^	105.8 (40.0)	108.9 (38.6)	102.6 (40.3)
HDL cholesterol mmol L^−1^	1.5 (0.4)	1.5 (0.4)	1.5 (0.4)
**Blood biomarkers**			
hsCRP mg L^−1^	1.7 (0.9;3.7)	1.6 (0.8;3.2)	1.6(0.9;4.0)
IL-6 pg mL^−1^	1.9 (1.4;2.8)	1.8 (1.3;2.7)	2.0 (1.4;3.3)
NT-proBNP ng L^−1^	149.0 (80.2;305.0)	136.0 (74.8;267.0)	177.5 (93.0;387.0)
hscTnT ng L^−1^	2.5 (2.5;9.1)	2.5 (2.5; 8.6)	5.8 (2.5;11.2)
hscTnI ng L^−1^	5.9 (4.4;9.0)	5.6 (4.3; 8.2)	6.6 (4.8;9.5)
**Lung function**			
FEV_1_ L	2.3 (0.7)	2.5 (0.6)	2.1 (0.6)
FVC L	3.3 (0.8)	3.3 (0.8)	3.3 (0.9)
FEV_1_/FVC	0.71 (0.09)	0.76 (0.05)	0.62 (0.07)
FEV_1_/FVC below 0.7 n (%)	338 (39.0%)	46 (10.7%)	338 (100%)
FEV_1_/FVC below LLN^c^ n (%)	270 (31.1%)	–	269 (79.6%)
Duration of follow-up years	7.48	7.67	7.12
Number of deaths	185	61	109
Mortality rate per 1,000 pyrs (95% CI)	28.5 (24.7; 32.9)	18.6 (14.4;23.9)	45.3 (37.6;54.7)

*pyrs* person years, *CI* confidence interval, *ETS* environmental tobacco smoke.

Data are mean (SD), n (%), or median (Q1, Q3) unless indicated otherwise.

^a^The information to define this respiratory healthy subpopulation was available for 813 of the 867 individuals, for definition of this population see methods section.

^b^Test was performed on a distance of 3 or 4 m, respectively, depending on space in the home of the participant.

^c^LLN was determined according to the reference equations by García-Río et al.^15^.

Compared to the 338 subjects with FEV_1_/FVC < 0.7, fewer subjects of the whole study population had a FEV_1_/FVC below LLN (n = 270, 31%), according to the reference equations from the older Spanish population^15^. 428 (53%) qualified as apparently lung healthy (out of 813 who had the information on lung health for the definition of this subgroup). Of those 428, 46 (10.8%) had an FEV_1_/FVC < 0.7.

### Lung function, biomarkers and mortality

The age and sex-adjusted correlation of lung function with biomarkers was inverse (Table 2) and ranged from − 0.04 (FEV_1_/FVC with hsCRP) to − 0.22 (FEV_1_ with hsCRP).

**Table 2:** Partial Spearman rank correlation coefficients for the relationship between lung function parameters and blood biomarkers after adjustment for age and sex.

	FEV_1_	FVC	FEV_1_/FVC	hsCRP	IL6	hscTnI	hscTnT
FEV_1_							
FVC	0.83						
FEV_1_/FVC	0.43	− 0.08					
High-sensitive C-reactive protein (hsCRP)	− 0.22	− 0.21	− 0.04				
Interleukin 6 (IL6)	− 0.19	− 0.17	− 0.06	0.52			
High-sensitive Troponin I (hscTnI)	− 0.12	− 0.10	− 0.07	0.16	0.26		
High-sensitive Troponin T (hscTnT)	− 0.15	− 0.12	− 0.08	0.16	0.24	0.47	
NT-proBNP	− 0.13	− 0.10	− 0.08	0.13	0.19	0.38	0.33

All correlations were statistically significant (*p* < 0.05), except for FEV_1_/FVC versus CRP (*p* = 0.144).

For the whole study population, the ratio FEV_1_/FVC was statistically significantly associated with all-cause mortality in all models. In the main model which included gait speed and daily walking time, the hazard ratio (HR) was 0.84 (95% CI 0.72–0.97) for an increase of 0.1 units of FEV_1_/FVC, and the HR was 1.55 (95% CI 1.14–2.11) for FEV_1_/FVC < 0.7 (Table 3). Notably, further adjustment neither for inflammatory nor for cardiac markers did alter the effect estimates much. The HR in the model including all functional as well as biomarkers was 0.86 (95% CI 0.74–0.99) in the whole study population for an increase of 0.1 units of FEV_1_/FVC and 1.49 (95% CI 1.09–2.03) for FEV_1_/FVC < 0.7. Comparable results were found for FEV_1_/FVC below LLN predicted. Analyses based on percent predicted lung function values showed the same pattern (Supplementary Table S1 online). The tests for interaction showed out of 20 tests only one p-value below 0.1, for hsCRP in relation with FEV_1_/FVC (results not shown), and may be considered a chance finding.

**Table 3:** Hazard ratios with 95% confidence intervals from Cox proportional hazards models^a^ evaluating the association between lung function and total mortality in the whole study population (N = 867).

Lung function measure	Base model^b^	Main model (MM)^c^	MM + hsCRP^d^ + IL-6^d^	MM + NT-proBNP^d^	MM + hscTnT^d^ + hscTnI^d^	MM + all biomarkers^e^
FEV_1_^f^	0.79 (0.68–0.91)	0.87 (0.74–*1.02*)	0.91 (0.77–*1.06*)	0.90 (0.76–*1.06*)	0.90 (0.76–*1.06*)	0.92 (0.78–*1.0*8)
FVC^f^	0.88 (0.78–*1.00*)	0.99 (0.87–*1.13*)	1.01 (0.89–*1.15*)	1.01 (0.88–*1.15*)	1.01 (0.88–*1.15*)	1.02 (0.90–*1.17*)
FEV_1_/FVC^g^	0.83 (0.73–0.95)	0.84 (0.72–0.97)	0.85 (0.74–0.99)	0.86 (0.74–0.99)	0.86 (0.74–0.99)	0.86 (0.74–0.99)
FEV_1_/FVC below 0.7	1.53 (1.13–2.07)	1.55 (1.14–2.11)	1.51 (1.11–2.06)	1.49 (1.09–2.03)	1.49 (1.09–2.03)	1.49 (1.09–2.03)
FEV_1_/FVC below LLN predicted	1.50 (1.11–2.01)	1.50 (1.10–2.05)	1.47 (1.07–2.01)	1.49 (1.09–2.03)	1.44 (1.06–1.97)	1.43 (1.04–1.95)

^a^All models adjust for age using age as the time-axis.

^b^Adjusted for sex and height.

^c^Adjusted for sex, height, current intake of respiratory medication, gait speed, daily walking time and HDL-cholesterol.

^d^As natural logarithm.

^e^hsCRP, IL-6, NT-proBNP, hscTnT, hscTnI (all natural log-transformed).

^f^Hazard ratio for an increase of 0.5 L.

^g^Hazard ratio for an increase of 0.1.

The effect estimates for FVC in the whole study population approached the null-effect value [0.99, 95% CI (0.87–1.13)] upon adjustment in the main model, whereas for FEV_1_ the HR was 0.87 (95% CI 0.74–1.02) for an increase of 0.5L (Tables 3 and 4), the latter attenuating to 0.92 (95% CI 0.78–1.08) after further adjustment for all biomarkers.

**Table 4:** Hazard ratios with 95% confidence intervals from Cox proportional hazards models^a^ evaluating the association between lung function and total mortality in the apparent lung healthy population (N = 428).

Lung function measure	Base model^b^	Main model (MM)^c^	MM + hsCRP^d^ + IL-6^d^	MM + NT-proBNP^d^	MM + hscTnT^d^ + hscTnI^d^	MM + all biomarkers^e^
FEV_1_^f^	0.77 (0.56–*1.05*)	0.82 (0.60–*1.14*)	0.89 (0.65–*1.22*)	0.84 (0.61–*1.17*)	0.82 (0.59–*1.14*)	0.88 (0.63–*1.22*)
FVC^f^	0.95 (0.74–*1.22*)	1.02 (0.79–*1.32*)	1.08 (0.84–*1.39*)	1.07 (0.82–*1.39*)	1.02 (0.79–*1.33*)	1.10 (0.85–*1.43*)
FEV_1_/FVC^g^	0.51 (0.31–0.84)	0.49 (0.29–0.81)	0.49 (0.30–0.82)	0.45 (0.26–0.76)	0.47 (0.28–0.79)	0.43 (0.25–0.73)
FEV_1_/FVC below 0.7	2.35 (1.32–4.17)	2.57 (1.44–4.59)	2.45 (1.36–4.42)	2.71 (1.51–4.86)	2.66 (1.48–4.78)	2.76 (1.52–4.93)

^a^All models adjust for age using age as the time-axis.

^b^Adjusted for sex and height.

^c^Adjusted for sex, height, current intake of respiratory medication, gait speed, daily walking time and HDL-cholesterol.

^d^As natural logarithm.

^e^hsCRP, IL-6, NT-proBNP, hscTnT, hscTnI (all natural log-transformed).

^f^Hazard ratio for an increase of 0.5 L.

^g^Hazard ratio for an increase of 0.1.

In the apparently lung healthy subpopulation, the estimated effects for FEV_1_/FVC were markedly stronger although less precise because of the smaller sample size (Table 4). For example, in the main model the HR was 0.49 (95% CI 0.29–0.81) versus 0.84 (95% CI 0.72–0.97) detected in the whole study population for 0.1 units increase of FEV_1_/FVC, and 2.57 (95% CI 1.44–4.59) versus 1.55 (95% CI 1.14–2.11) for FEV_1_/FVC < 0.7. As in the whole study population, the effect estimates were robust to adjustment for inflammatory or cardiac markers. Effect estimates for FVC and FEV_1_ in apparently lung healthy subjects were comparable to those observed for the whole study population.

In both, the whole study population and the lung healthy subpopulation, models were robust to adjustment for smoking status (which did however, not qualify for inclusion into our models—details see methods) in a sensitivity analysis (see Supplementary Tables S2 and S3 online). Including individuals with only one acceptable spirometry curve or a reproducibility > 0.2L (N = 1,032) did not change the overall pattern of the results (data not shown).

## Discussion

In our population-based study of relatively healthy older adults, we found a robust statistically significant association with 10-year all-cause mortality for the ratio FEV_1_/FVC, with a possible tendency for FEV_1_, but no association for FVC.

Adjustment for inflammatory and specific cardiac biomarkers in addition to other functional parameters (i.e. gait speed and sensor based-PA) did not alter effect estimates markedly. A comparable pattern of effect estimates but markedly stronger was observed in the apparently lung health subpopulation. These results support the role of FEV_1_/FVC as an independent predictor of mortality in older adults beside other specific and well-characterized biomarkers.

### Lung function and mortality

In general and middle-aged populations, studies found an association of FEV_1_ with mortality^19–21^, and partly showed that FEV_1_ was a better predictor than FVC^22^, while other studies reported a stronger association with FVC and less (or no) association with FEV_1_ or airway obstruction^1–3^. In older populations, an association with FEV_1_^23^ and airway obstruction with all‐cause mortality^24^ has been reported. We observed also a clear association for FEV_1_/FVC. However, we found an association with FVC only in the crude model. After multivariable adjustment, which included gait speed and daily walking time, factors rarely adjusted for in other studies^1,3^, the association was attenuated to 0.99 and became non-significant.

The association of airflow limitation with mortality was even stronger in healthy individuals, excluding current smokers, which might, at least partly, be due to a relatively low baseline absolute risk in this subpopulation. Respective findings for non-smokers are heterogeneous: some investigators^19^ found an association with FEV_1_, FVC and FEV_1_/FVC in middle aged subjects, while others^25^ only studying FEV_1_ found none [HR 1.01 (95% CI 0.91–1.11) for 10% decrease in predicted]. Similarly to the last named study^25^ we adjusted for physical function but still found an association. These findings in in asymptomatic, apparently lung healthy older adults suggest the importance of performing lung function tests also for these individuals.

### Relation with inflammation

In general, IL-6 and hsCRP concentrations increase with age^26–29^, so that the term “inflamm-aging” has been coined^30^. Moreover, Puzianowska et al. reported that IL-6 and hsCRP levels were associated with decreased physical and cognitive performance and increased mortality risk in an older population, even in those called successfully aging individuals^27^. COPD and reduced FEV_1_ have also been associated with higher levels of hsCRP and IL-6 amongst other inflammatory markers in the general population as well as among older adults^10,31,32^. Note, however, that systemic inflammation is not always detectable in COPD patients^33^. Airflow limitation and COPD, as well as diseases like CVD and diabetes share chronic low grade systemic inflammation, which may explain part of the relationship of lung function with total mortality and not only with respiratory mortality. In addition, the relation between reduced lung function and stroke may be explained by systemic inflammation^34^.

This common link of different chronic disease with systemic inflammation may be due to common risk factors such as smoking, lack of PA or ambient air pollution, which are known to influence oxidative stress and inflammation^35,36^. It is hypothesized that inflammation in the lung, caused by external exposures, such as smoke or air pollution, or being present in chronic lung disease, may “spill over” via inflammatory cells and pro-inflammatory mediators leading to systemic inflammation^36–38^.

Inflamm-aging and its influence on chronic disease, including COPD, and frailty in the older population has been postulated^30^. Therefore, one could expect that the association of airflow limitation with mortality is attenuated upon adjustment for inflammatory markers, especially in older individuals. In the Whitehall II Cohort Study on civil-servants aged ≥ 60 years the HR for the association of mortality with FEV_1_^23^ from a base model adjusted for smoking was reduced by 21% (from 1.86 to 1.63) upon further adjustment by hsCRP and IL-6. This change was larger than that by adjustment for other life-style or cardiovascular risk factors or for comorbidities. In our analyses, however, the HRs changed only little after adjustment for IL-6 or hsCRP. The association between air-flow limitation and total mortality in older adults could be explained through an increased ventilation inhomogeneity in the lungs, which causes increased work of breathing leading to increased oxygen consumption by respiratory muscles. On a systemic level, ventilation inhomogeneity could result in lower oxygen saturation and pressure and, hence, would decrease the amount of oxygen delivered to the different organs and tissue areas. Together with physical exertion, organ stress or other challenging factors, this may lead to regional or global hypoxia increasing the risk for fatal events. In order to get a deeper insight in the involved pathophysiological mechanisms more data from older populations is needed.

### Relation with cardiac markers

In our study population, the association between airflow limitation and mortality was not influenced by markers of cardiac stress (NT-proBNP) or myocardial injury (troponins). Elevated levels of these cardiac markers have been reported in patients with COPD^39,40^ and are clearly associated with mortality as shown in an earlier analysis from the ActiFE-study^41^.

Strong inverse associations of FEV_1_ and FVC with hscTnT and NT-proBNP have been found in a general population of older men^10^. Magnussen et al.^1^ found an association of FEV_1_ and FVC with overall mortality irrespective of adjustment for these cardiac markers and HF, and no interaction between lung function and HF^1^. They concluded that cardiac dysfunction is not driving the association between lung function and mortality in their general population including all ages up to 74 years old individuals. We extend these findings by showing that, in a general older population, the effect of airflow limitation (measured as FEV_1_/FVC), on mortality seem not to be influenced by markers of cardiac dysfunction.

### Strengths and limitations

Our study consists of a large sample of community-dwelling older adults that also includes a high number of very old subjects. Nevertheless, the sample size might still have been too small to appropriately evaluate the association with all parameters measured by the spirometry.

Participants have been characterised in detail regarding established risk factors and functional ability. Information on comorbidities were only available by self-report. However, a validation study carried out during the three years of follow-up of the same cohort among 223 subjects, showed that the diagnoses of diabetes mellitus, stroke and heart attack could be confirmed by general practitioners (GP) for 96.3%, 76.0% and 72.9% of the cases, respectively. This shows that major diseases are in general accurately reported by the patients, in line with published literature^42^.

PA was measured objectively and, in addition to walking speed, proved to be a crucial covariate in our analysis. We have to acknowledge, however, that the initially relatively low participation rate of 20%^43^ may reflect a self-recruitment of predominantly healthier and fitter individuals into the study. Prevalence of pulmonary diseases such as asthma (5.5%) and COPD (2.6%) were noted to be low when compared to data from a survey of primary care services in Germany indicating that approximately 8% and 14% of older female and male patients, respectively, were diagnosed with chronic bronchitis, emphysema or chronic airway obstruction^44^. However, our estimates of frailty, hypertension and diabetes prevalence are in concordance with the available literature^45^. Given that our population is relatively healthy, and perhaps has a lower mortality risk, our observed results with respect to airflow limitation are even more remarkable and point out the possible role of spirometry for the identification of older individuals at high mortality risk. Similarly, individuals in our final analysis had a lower mortality rate compared to the 1506 individuals recruited at baseline. This may also explain why smoking did not qualify as an important confounding risk factor, as only 5.9% were current smokers. Nevertheless, the low response rate should not affect the internal validity of our findings in the follow-up part of our study. In addition, we do not have information on causes of death precluding a more in depth analysis with regard to respiratory and cardiovascular deaths and the importance of biomarkers therein.

In conclusion, our study in a large cohort of community-dwelling older adults showed that airflow limitation as measured by FEV_1_/FVC is linked to all-cause mortality independently of markers of inflammation and cardiac stress and injury in a model that also accounted for markers of physical activity and functionality. The association for airflow limitation was even stronger in non-smoking apparently lung healthy individuals. Therefore, pulmonary function tests appear to deliver additional information beyond cardiac and functional parameters and allow for more confident risk stratification in older adults. Future studies that can investigate cause-specific mortalities would be highly useful to understand which diseases are most relevant for lung function predicted mortality irrespective of inflammation and cardiac markers.

## Supplementary information

Supplementary information

## Data Availability

Due to ethical restrictions regarding data protection issues and the study specific consent text and procedure, the data cannot be made publicly available, but data are available to all interested researchers upon request.
