# Supplementary material for: Association of lung function with overall mortality is independent of inflammatory, cardiac, and functional biomarkers in older adults: the ActiFE-study
Source: Sci Rep. 2020 Jul 17;10:11862. doi: 10.1038/s41598-020-68372-w (PMC7367870; doi:10.1038/s41598-020-68372-w)
Supplement: Supplementary file 1 — Supplementary information [file 41598_2020_68372_MOESM1_ESM.docx]

Supplementary Information online for:

Association of lung function with overall mortality is independent of inflammatory, cardiac, and functional biomarkers in older adults: ActiFE-study

Gudrun Weinmayr^1*^, Holger Schulz^2,3^, Jochen Klenk^1,4,5^, Michael Denkinger^6,7^, Enric Duran-Tauleria^8^, Wolfgang Koenig^1,9,10^, Dhayana Dallmeier^6,7,11**^/Dietrich Rothenbacher^1**^, the ActiFE study group.

** both authors contributed equally

^1^ Institute of Epidemiology and Medical Biometry, Ulm University, Ulm, Germany

^2^Institute of Epidemiology, Helmholtz Zentrum München – German Research Center for Environmental Health, Neuherberg, Germany

^3^Comprehensive Pneumology Center Munich (CPC-M), Member of the German Center for Lung Research (DZL), Munich, Germany

^4^ Department of Geriatrics and Geriatric Rehabilitation, Robert-Bosch-Hospital, Stuttgart, Germany

^5^ IB Hochschule Berlin, Studienzentrum Stuttgart, Stuttgart, Germany

^6^ Agaplesion Bethesda Hospital, Geriatric Research Unit, Ulm University

^7^Geriatric Center Ulm/Alb-Donau, Ulm, Germany

^8^ Global Institut of Neurodevelopment Integrated Care (IGAIN), Barcelona, Spain

^9^ Deutsches Herzzentrum München, Technische Universität München, Munich, Germany

^10^ DZHK (German Centre for Cardiovascular Research), partner site Munich Heart Alliance, Technische Universität München, Munich, Germany

^11^ Dept. of Epidemiology, Boston University School of Public Health, Boston, USA

***Corresponding author:**

Gudrun Weinmayr

Institute of Epidemiology and Medical Biometry

Ulm University

Helmholtzstraße 22

89081 Ulm

Germany

E-Mail: [Gudrun.Weinmayr@uni-ulm.de](mailto:Gudrun.Weinmayr@uni-ulm.de)

Contents:

- Detailed description of the methods
- Supplementary Table S1. Association between lung function and total mortality in the whole study population (N=867): lung function variables based on reference equations
- Supplementary Table S2. Sensitivity analysis: additional adjustment for smoking status of the association between lung function and total mortality in the whole study population (N=867)
- Supplementary Table S3. Sensitivity analysis: additional adjustment for smoking status (ex-smoker; never smoker) of the association between lung function and total mortality in the apparent lung healthy population (N=426)

Detailed description of the methods:

***Study population:***

The study protocol of the Activity and Function in the Elderly (ActiFE) Ulm study ^1^, including details of recruitment and the study population have been reported in details elsewhere ^2,3^. In brief, a population- based random sample of community-dwelling older adults (≥ 65 years) in Ulm and adjacent areas were contacted by mail. Of the eligible persons (inclusion criteria were no severe cognitive, auditive or visual deficits and no serious German language difficulties), 1506 individuals participated.

Participants underwent an extensive standardized baseline interview and examination between March 2009 and April 2010 and were asked to wear a uni-axial accelerometer (activPAL, PAL Technologies, Glasgow, Scotland) for 7 consecutive days (24h per day) to measure PA (details in ^2^). In addition, a blood sample was drawn. All participants provided written informed consent and the Ethics Committee of the University of Ulm had approved the study (application no. 318/08 and 50/12).

***Laboratory measurements***

Blood was drawn under standardized conditions, centrifuged, aliquoted and stored at -80°C. C-reactive protein (hsCRP) was measured with a high- sensitivity assay on a Behring Nephelometer II (interassay CV 5.2%–6.4%). Interleukin-6 (IL-6) was measured by ELISA with ELISA Quantikine HS,R&D range 0,156-10 pg/ml (inter-assay CV < 10%). NT-proBNP was measured by electrochemoluminescence on an Elecsys 411 (Roche Diagnotics) (coefficient of variation (CV) < 5%, lower limit of detection (LOD) of 5 ng/L). High-sensitive troponin I (hscTnI) was measured on an ARCHITECT STAT (Abbott Diagnostics), with a within-laboratory imprecision of ≤ 10% CV across the range of 10 to 50000 ng/L (LOD equal to 2.0 ng/L), and a reported within-run and within-laboratory CV < 5.5%. HscTnT was also measured on an Elecsys 411 with a LOD equal to 5 ng/L. Total cholesterol and HDL cholesterol (homogeneous method) were measured from unfrozen samples the same day of blood draw spectrophotometrically in a Dimension RxL Max Integrated Chemistry System (Siemens) on the basis of a reaction catalyzed by cholesterol oxidase, with Siemens reagents. All markers were measured in a blinded fashion.

***Statistical analysis***

Characteristics of the study population were calculated for the whole study population as well as for subjects with FEV_1_/FEVC <0.7 and ≥0.7, respectively. In addition, we displayed main characteristics for a defined respiratory healthy population (details see below). Correlations between lung function parameters and clinical markers were assessed with Spearman rank correlation coefficients adjusted for age and sex.

Cox-proportional hazards regression models were used to estimate the association of various lung function parameters with eight-year mortality. Models were fitted with age as the underlying time variable ^4^. FEV_1_, FVC and FEV_1_/FVC were incorporated as continuous variables and all models adjusted for sex and height in addition to age. Furthermore, a dichotomous variable with cutpoint 0.7 was used for FEV_1_/FVC. As there is debate whether this fixed cutpoint is appropriate for the older population we also used percent predicted values based on equations of Karrasch et al ^5^ and García-Río et al ^6^ in a sensitivity analysis. The former population is geographically close to Ulm (about 80 km away) but includes also individuals younger than our study population. The second study population includes specifically older adults >65 years and is located in Barcelona, Spain.

In addition, we analysed the association of lung function parameters, cardiac markers and further covariates with overall mortality in the apparent lung healthy part of the study population which was defined as follows: FEV_1_/FVC ≥ lower limit of normal (LLN) of predicted value, no self-reported diagnoses of COPD, asthma, chronic bronchitis or emphysema, no self-reported respiratory symptoms (wheeze, cough and phlegm over at least 3 months per year), no self-reported current intake of respiratory medication, no current smoker. The information to define this population was available for 813 of the 867 individuals. FEV_1_/FVC ≥ LLN was chosen rather than the fixed ratio due to its higher specificity in an older population ^7^ to retain most of the healthy individuals for this analysis.

*Model development*

Based on previous literature and an informal directed acyclic graph, the following covariates were investigated for empirical model development: duration of school education (≤ 9 years vs. >9 years (as proxy for socioeconomic status)), body mass index (BMI) (kg/m^2^, continuous), weight (in kg, continuous), sensor-based PA measured as daily walking time (minutes/day), smoking status (current, ex-, never smoker), ETS (“Has anyone living in your home (besides yourself) smoked a cigarette, pipe or cigar in your home during the past two weeks? “ yes/no), self-reported occupational exposure to dust (“Have you ever worked for a year or more in a dusty job? “)and chemicals (“Have you ever worked for a year or more exposed to fumes, gases or chemicals?” yes/no) and traffic exposure at home (cars or heavy vehicles pass frequently or constantly the house vs. never or seldom), cholesterol (including HDL and LDL cholesterol, all in mmol/L), self-reported current intake of respiratory medication (“Do you currently take any medication or sprays to facilitate breathing?”) handgrip strength (kg) and gait speed in m/s. Gait speed was measured either on a 3m or a 4m distance, depending on the space that was available in the participants home. The continuous variable gait speed was standardised by calculating the speed as m/s using standardization factors.

These variables were investigated for their association with mortality. Factors that were not related to mortality (p<0.05) were not further considered except for education and respiratory medicine. If several variables were indicators for a same underlying concept, the one with the strongest association was retained. E.g. weight and not BMI was investigated further. The thus identified factors entered then the stepwise forward selection process to yield a parsimonious model: only those covariates that caused a change in the log hazard ratio by 5% or more for any of the three continuous exposures (FEV_1_ FVC FEV_1_/FEVC) were retained. 5% was chosen as a more conservative approach than the usual 10%.

This selection procedure yielded the main model incorporating gait speed, daily walking time, intake of respiratory medicine and HDL-cholesterol in addition to sex, age and height.

Deviation from proportional hazards assumption in the Cox model was assessed by testing for a non-zero slope in a generalized linear regression of the scaled Schoenfeld residuals on time ^8^

To this final main model, clinical biomarkers (NT-proBNP, troponins, hsCRP and IL-6) were introduced after log-transformation (natural log). We also tested for interaction of the biological markers with the lung function parameters by introducing an interaction term into the model. All analyses were conducted using Stata software (version 14; StataCorp, College Station, TX, USA).

References

1. Denkinger, M. D. *et al.* Accelerometer-based physical activity in a large observational cohort--study protocol and design of the activity and function of the elderly in Ulm (ActiFE Ulm) study. *BMC Geriatr.* **10,** 50 (2010).

2. Klenk, J. *et al.* Objectively Measured Walking Duration and Sedentary Behaviour and Four-Year Mortality in Older People. *PLoS One* **11,** e0153779 (2016).

3. Dallmeier, D. *et al.* A prospective assessment of cardiac biomarkers for hemodynamic stress and necrosis and the risk of falls among older people: the ActiFE study. *Eur. J. Epidemiol.* **31,** 427–35 (2016).

4. Thiébaut, A. C. M. & Bénichou, J. Choice of time-scale in Cox’s model analysis of epidemiologic cohort data: a simulation study. *Stat. Med.* **23,** 3803–3820 (2004).

5. Karrasch, S. *et al.* Spirometric reference values for advanced age from a South german population. *Respiration.* **85,** 210–9 (2013).

6. García-Río, F., Pino, J. M., Dorgham, A., Alonso, A. & Villamor, J. Spirometric reference equations for European females and males aged 65-85 yrs. *Eur. Respir. J.* **24,** 397–405 (2004).

7. Oh, D. K. *et al.* Comparison of the fixed ratio and the Z-score of FEV1/FVC in the elderly population: a long-term mortality analysis from the Third National Health and Nutritional Examination Survey. *Int. J. Chron. Obstruct. Pulmon. Dis.* **13,** 903–915 (2018).

8. Grambsch, P. M. & Therneau, T. M. Proportional hazards tests and diagnostics based on weighted residuals. *Biometrika* **81,** 515–526 (1994).

**Supplementary Table S1**. Hazard ratios with 95% confidence intervals from Cox proportional hazards models^a^ evaluating the association between lung function and total mortality in the whole study population (N=867): lung function variables based on reference equations

|  | Base Model^b^ | Main Model^c^ (MM) | MM + all biomarkers^d^ |
| --- | --- | --- | --- |
| Reference equations according to ^5^ | | | |
| FEV_1_ (% of predicted) ^e^ | 0.93 (0.89;0.97) | 0.95 (0.91;0.99) | 0.96 (0.92;1.01) |
| FVC (% of predicted) ^e^ | 0.95 (0.91;0.99) | 0.98 (0.94;1.03) | 0.99 (0.95;1.04) |
| FEV_1_/FVC (% of predicted) ^e^ | 0.93 (0.89;0.98) | 0.93 (0.88;0.98) | 0.94 (0.89;1.00) |
| FEV_1_/FVC below LLN predicted | 1.52 (1.01;2.29) | 1.31 (0.84;2.05) | 1.25 (0.80;1.96) |
| Reference equations according to ^6^l | | | |
| FEV_1_ (% of predicted) ^e^ | 0.94 (0.91;0.97) | 0.95 (0.92;0.99) | 0.96 (0.93;1.00) |
| FVC (% of predicted) ^e^ | 0.96 (0.92;0.99) | 0.98 (0.94;1.01) | 0.98 (0.95;1.02) |
| FEV_1_/FVC (% of predicted) ^e^ | 0.94 (0.89;0.98) | 0.93 (0.88;0.99) | 0.94 (0.89;1.00) |
| FEV_1_/FVC below LLN predicted | 1.50 (1.11;2.01) | 1.50 (1.10;2.05) | 1.43 (1.04;1.95) |

a: all models adjust for age using age as the time-axis; b: adjusted for sex and height; c: adjusted for sex, height, current intake of respiratory medicine,

gait speed, daily walking time and HDL-cholesterol; d: hsCRP, IL-6, NT-proBNP, hscTnT, hscTnI (all natural log-transformed) ; e: hazard ratio for an increase of 5%

References:

1. Denkinger, M. D. *et al.* Accelerometer-based physical activity in a large observational cohort--study protocol and design of the activity and function of the elderly in Ulm (ActiFE Ulm) study. *BMC Geriatr.* **10,** 50 (2010).

2. Klenk, J. *et al.* Objectively Measured Walking Duration and Sedentary Behaviour and Four-Year Mortality in Older People. *PLoS One* **11,** e0153779 (2016).

3. Dallmeier, D. *et al.* A prospective assessment of cardiac biomarkers for hemodynamic stress and necrosis and the risk of falls among older people: the ActiFE study. *Eur. J. Epidemiol.* **31,** 427–35 (2016).

4. Thiébaut, A. C. M. & Bénichou, J. Choice of time-scale in Cox’s model analysis of epidemiologic cohort data: a simulation study. *Stat. Med.* **23,** 3803–3820 (2004).

5. Karrasch, S. *et al.* Spirometric reference values for advanced age from a South german population. *Respiration.* **85,** 210–9 (2013).

6. García-Río, F., Pino, J. M., Dorgham, A., Alonso, A. & Villamor, J. Spirometric reference equations for European females and males aged 65-85 yrs. *Eur. Respir. J.* **24,** 397–405 (2004).

7. Oh, D. K. *et al.* Comparison of the fixed ratio and the Z-score of FEV1/FVC in the elderly population: a long-term mortality analysis from the Third National Health and Nutritional Examination Survey. *Int. J. Chron. Obstruct. Pulmon. Dis.* **13,** 903–915 (2018).

8. Grambsch, P. M. & Therneau, T. M. Proportional hazards tests and diagnostics based on weighted residuals. *Biometrika* **81,** 515–526 (1994).

**Supplementary Table S2**. Sensitivity analysis: additional adjustment for smoking status. Hazard ratios with 95% confidence intervals from Cox proportional hazards models^a^ evaluating the association between lung function and total mortality in the whole study population (N=867)

| Lung function measure | Main Model (MM)^c^ | MM + hsCRP^d^ + IL-6^d^ | MM +  NT-proBNP^d^ | MM +  hscTnT^d^ +  hscTnI^d^ | MM + all biomarkers^e^ |
| --- | --- | --- | --- | --- | --- |
| FEV_1_^f^ | 0.87 (0.74;1.02) | 0.91 (0.77;1.07) | 0.90 (0.76;1.06) | 0.91 (0.77;1.07) | 0.92 (0.78;1.09) |
| FVC ^f^ | 0.99 (0.87;1.13) | 1.02 (0.89;1.16) | 1.01 (0.88;1.16) | 1.02 (0.89;1.16) | 1.03 (0.90;1.17) |
| FEV_1_/FVC ^g^ | 0.84 (0.72;0.97) | 0.85 (0.74;0.99) | 0.85 (0.74;0.99) | 0.85 (0.74;0.99) | 0.86 (0.74;0.99) |
| FEV_1_/FVC below 0.7 | 1.56 (1.14;2.13) | 1.52 (1.11;2.08) | 1.50 (1.10;2.04) | 1.52 (1.11;2.08) | 1.49 (1.09;2.04) |
| FEV_1_/FVC below LLN predicted | 1.51 (1.11;2.06) | 1.47 (1.08;2.02) | 1.45 (1.06;1.98) | 1.47 (1.08;2.02) | 1.43 (1.04;1.96) |

a: all models adjust for age using age as the time-axis; b: adjusted for sex and height; c: adjusted for sex, height, current intake of respiratory medicine, gait speed, daily walking time and HDL-cholesterol; d: as natural logarithm; e: hsCRP, IL-6, NT-proBNP, hscTnT, hscTnI (all log-transformed); f: hazard ratio for an increase of 0.5 L; g: hazard ratio for an increase of 0.1;

**Supplementary Table S3**. Sensitivity analysis: additional adjustment for smoking status (ex-smoker; never smoker) Hazard ratios with 95% confidence intervals from Cox proportional hazards models^a^ evaluating the association between lung function and total mortality in the apparent lung healthy population (N=426)

| Lung function measure | Main Model (MM)^c^ | MM + hsCRP^d^ + IL-6^d^ | MM +  NT-proBNP^d^ | MM +  hscTnT^d^ +  hscTnI^d^ | MM + all biomarkers^e^ |
| --- | --- | --- | --- | --- | --- |
| FEV_1_^f^ | 0.82 (0.59;1.14) | 0.89 (0.64;1.22) | 0.84 (0.60;1.17) | 0.81 (0.58;*1.13*) | 0.88 (0.63;*1.22*) |
| FVC ^f^ | 1.03 (0.80;1.34) | 1.08 (0.84;1.39) | 1.07 (0.82;1.39) | 1.03 (0.79;*1.34*) | 1.10 (0.85;*1.43*) |
| FEV_1_/FVC ^g^ | 0.44 (0.26;0.75) | 0.46 (0.28;0.78) | 0.42 (0.24;0.72) | 0.43 (0.25;0.7380) | 0.41 (0.24;0.71) |
| FEV_1_/FVC below 0.7 | 2.73 (1.52;4.90) | 2.55 (1.40;4.64) | 2.81 (1.56;5.05) | 2.79 (1.54;5.03) | 2.80 (1.53;5.15) |

a: all models adjust for age using age as the time-axis; b: adjusted for sex and height; c: adjusted for sex, height, current intake of respiratory medicine, gait speed, daily walking time and HDL-cholesterol; d: as natural logarithm; e: hsCRP, IL-6, NT-proBNP, hscTnT, hscTnI (all log-transformed); f: hazard ratio for an increase of 0.5 L; g: hazard ratio for an increase of 0.1;
